# TIMP1 as a context-dependent biomarker linking cancer progression and cardiovascular disorders: a multi-level and bioinformatics study

**DOI:** 10.3389/fimmu.2025.1699962

**Published:** 2026-01-14

**Authors:** Haomin Xie, Dianhua Zhou, Sicheng Chen, Yining Dai, Ranran Zhang, Zongrong Lin, Weiyi Kong, Jianfang Luo, Zhenyang Fu

**Affiliations:** 1 Department of Cardiovascular Disease, Shantou Hospital of Traditional Chinese Medicine, Shantou, China; 2 The First Clinical Medical School of Guangdong Pharmaceutical University, Guangzhou, China; 3 Department of Cardiology, Guangdong Provincia People’s Hospital (Guangdong Academy of Medical Sciences), Southern Medical University, Guangzhou, China; 4 Faculty of Health, Medicine and Behavioral Sciences, The University of Queensland, Brisbane, Australia; 5 Stomatological Hospital, School of Stomatology, Southern Medical University, Guangzhou, China; 6 Department of Cardiology, The First Affiliated Hospital of Guangdong Pharmaceutical University, Guangdong Pharmaceutical University, Guangzhou, China

**Keywords:** cardiovascular disease, colorectal cancer, gastric cancer, immune microenvironment, Pan-cancer, TIMP1

## Abstract

**Background:**

Tissue inhibitor of metalloproteinase 1 (TIMP1) plays diverse roles in extracellular matrix (ECM) remodeling, immune regulation, and tumor progression. However, its systemic patterns across cancers and cardiovascular disease remain incompletely understood.

**Methods:**

We applied an integrative pipeline beginning with microarray analysis of tumor-bearing mouse hearts (GSE63032) to identify TIMP1 as a hub gene. Pan-cancer datasets from TCGA/GTEx and public portals were analyzed for expression, genomic alterations, epigenetic regulation, immune infiltration, prognosis, and drug sensitivity. Single-cell RNA-seq was used to define cell type–specific expression. As all large-scale analyses were performed using publicly available bioinformatics datasets, cell line experiments were used for targeted validation of TIMP1 expression and function. Findings were validated by immunohistochemistry, western blotting, and transwell assays in colorectal and gastric cancer models, with additional analysis performed in atherosclerosis cohort (GSE100927) and heart failing cohort (GSE5406) to explore cardiovascular relevance.

**Results:**

TIMP1 was consistently upregulated across cancers, especially in colorectal and gastric tumors, where it correlated with adverse survival and high diagnostic accuracy. Genomic analyses revealed copy number alterations, while promoter hypomethylation aligned with increased expression in digestive cancers. Drug-response profiling indicated sensitivity to epigenetic inhibitors and resistance to MAPK-targeted agents. Single-cell analyses localized TIMP1 to myeloid cells in colorectal cancer and fibroblasts in gastric cancer, linking it to apoptosis, EMT, angiogenesis, and stromal–immune crosstalk. Beyond oncology, TIMP1 was elevated in atherosclerosis, aligning with immune- and lipid-related pathways, but reduced in heart failure, where it was linked to impaired mitochondrial metabolism.

**Conclusion:**

This multi-level and bioinformatics study identifies TIMP1 as a cross-disease regulator with context-dependent functions. TIMP1 serves as a potential prognostic and diagnostic biomarker in digestive cancers, a therapeutic stratification marker for epigenetic interventions, and a candidate mediator linking tumor biology with cardiovascular disorders such as atherosclerosis and heart failure.

## 1 Introduction

Tissue inhibitor of metalloproteinase 1 (TIMP1) is a multifunctional glycoprotein that plays a central role in extracellular matrix (ECM) remodeling, metalloproteinase inhibition, and immune regulation. Beyond its canonical role in matrix homeostasis, TIMP1 has been implicated in diverse biological processes including cell proliferation, apoptosis, and cytokine signaling. These features make TIMP1 a molecule of considerable interest in both oncology and cardiovascular research, where ECM remodeling and immune dysregulation are hallmarks of disease progression (1).

In cancer biology, elevated TIMP1 expression has been documented in several malignancies, particularly colorectal, gastric, and breast cancers, where it correlates with tumor invasion, metastasis, and fibrosis. TIMP1 has also been associated with adverse prognosis in multiple cohorts, often reflecting its role in shaping the tumor microenvironment through fibroblast activation and immune modulation (2). In cardiovascular disease, TIMP1 has been studied in the context of atherosclerosis, myocardial fibrosis, and heart failure, where it is linked to inflammatory signaling and ECM turnover (3). These findings collectively suggest that TIMP1 may function as a regulator at the intersection of tumor biology and cardiovascular remodeling.

Despite these observations, important gaps remain. Prior studies have largely focused on individual cancer types or single disease models, without providing a comprehensive overview of TIMP1 across malignancies. The cross-disease implications of TIMP1, particularly its roles in linking tumor-derived stress responses to cardiovascular remodeling, remain poorly understood. Furthermore, most available studies are limited to single-layer analyses, lacking integration across transcriptomic, genomic, epigenetic, and immune dimensions, and seldom coupled with functional validation (4).

To address these gaps, we designed a pan-cancer, multi-level, and bioinformatics study to systematically characterize TIMP1. Using integrative transcriptomic, genomic, epigenetic, immunological, and pharmacogenomic analyses, we evaluated TIMP1 across cancer types and extended the investigation to atherosclerosis and heart failure as representative cardiovascular disorders. Functional assays in colorectal and gastric cancer models were further performed to validate the oncological significance of TIMP1. Through this framework, we aimed to establish TIMP1 as a context-dependent regulator and candidate biomarker at the interface of tumor progression, immunity, and cardiovascular pathology.

## 2 Materials and methods

We implemented a four-stage pipeline: (i) GSE63032 microarray preprocessing, PCA, and *limma*-based DEGs (|logFC|≥1, FDR<0.05); (ii) network prioritization (*STRING/MCODE*) nominating TIMP1; (iii) pan-cancer characterization (expression; TMB/MSI; CNV/mutation; immune infiltration; survival/diagnostic performance; GO/KEGG & GSEA; scRNA-seq and cardiovascular validations; drug–response screens); and (iv) HPA subcellular/IHC and experimental validation (WB, Transwell).

### 2.1 Data acquisition

First, to identify core hub genes linked to cancer-cardiovascular crosstalk, we retrieved the microarray dataset GSE63032 (heart tissue from C26 tumor-bearing mice, n=3; non-tumor-bearing controls, n=3) (5) from GEO, performed data preprocessing, analyzed differential gene expression using the *limma* package (defining DEGs with |logFC|≥1 and FDR<0.05), conducted GO/KEGG enrichment analysis for DEGs, constructed a PPI network via *STRING*, and screened hub genes using *MCODE* to prioritize TIMP1. Missing values (<1% of the matrix) were excluded.

Next, to systematically characterize TIMP1 across cancers, we utilized pan-cancer datasets from *TCGA/GTEx*, *cBioPortal* and *ICGC*: we analyzed TIMP1 expression patterns, genomic alterations (copy number variations and mutations), epigenetic regulation (promoter methylation via *UALCAN/GSCA*), immune cell infiltration (#via *XCELL/TIMER2*), prognostic and diagnostic value (Cox regression, Kaplan-Meier survival, and ROC curve analyses), cell type-specific expression (single-cell RNA-seq of COAD [GSE146771] and STAD [GSE134520] datasets), and drug sensitivity (using GDSC/CTRP data) to explore its potential as a therapeutic stratification marker.

Subsequently, to investigate TIMP1’s role in cardiovascular disorders, we analyzed two GEO datasets: GSE100927 (atherosclerosis) and GSE5406 (heart failure), performing differential expression analysis, ROC curve analysis, and GSEA/GO/KEGG enrichment to link TIMP1 to relevant pathways.

**Figure 1:**
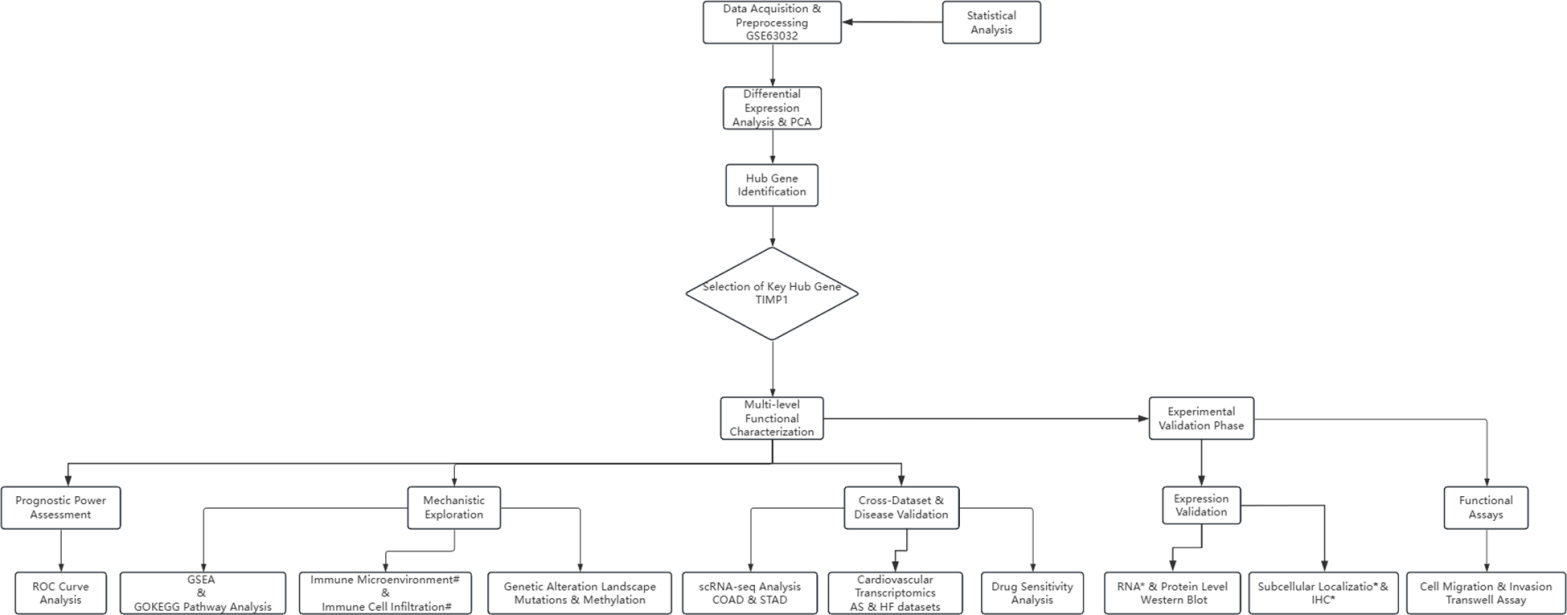
Overview of the analytical workflow. An integrative bioinformatics pipeline was constructed to evaluate TIMP1 across cancers. (*Data were from *HPA* or *TCGA*) (#Data were from *XCELL*).

Finally, to validate TIMP1’s expression and functional role in COAD and STAD, we conducted immunohistochemical (IHC) staining to compare protein levels in tumor and normal tissues(*Data were from *HPA* or *TCGA*), Western blotting to quantify TIMP1 protein in cancer cell lines (HCT116/SW480/LoVo for COAD; AGS/MKN45/SGC7901 for STAD) versus normal cell lines (FHC for COAD; GES-1 for STAD), and Transwell assays with TIMP1 overexpression, knockdown, and empty vector control groups to evaluate its impact on cancer cell migration and invasion (**Figure 1**).

### 2.2 Differential expression analysis and principal component analysis

Differential gene expression was analyzed using the *limma* package in R (6). Genes with |logFC| ≥ 1 and false discovery rate (FDR; Benjamini–Hochberg) < 0.05 were defined as differentially expressed genes (DEGs). Heatmaps were generated using the *pheatmap* package (v1.0.12), and PCA was performed using the *prcomp* function in R. Unless otherwise specified, in-house analyses report FDR-controlled results (|logFC|≥1, adj.p<0.05).

### 2.3 Hub gene analysis

Hub genes were identified based on topological analysis of PPI networks and intersected with DEGs. Genes that intersected were prioritized based on degree centrality from the *STRING* (7) network. Functional similarity was evaluated using the *MCODE* (8), and a similarity network was constructed to refine hub gene selection.

### 2.4 Data sources and processing

Open-access datasets were obtained from The Cancer Genome Atlas *(TCGA*; https://portal.gdc.cancer.gov/*)(log2(TPM + 1))*, *Genomic Data Commons (GDC)*. RNA-seq data were normalized via the *edgeR* (9) package (R), and data integrity was validated using Bioconductor tools. For results derived from public web portals (e.g., *TIMER2*, *TISIDB*, *UALCAN*, *GSCA*, *cBioPortal*), the platforms’ reported nominal p-values are used and interpreted as exploratory (see Statistical Analysis).

### 2.5 RNA and protein expression analysis

RNA expression and protein abundance across human tissues were queried from the *Human Protein Atlas* (*HPA;*https://www.proteinatlas.org/) and *TCGA*. All tissue-level results were directly extracted from the HPA interface, which provides values only in graphical rather than tabular format; therefore, RNA and protein profiles are presented solely as visual outputs in the manuscript. Tissue-specific TIMP1 signatures (focusing on oncological specimens) were statistically evaluated; inter-tissue protein variability was assessed via one-way *ANOVA*, with visualization using *ggplot2* (R) (10).

### 2.6 Gene expression analysis

TIMP1 transcriptomic profiling was performed using *TCGA RNA-seq data* (log2(TPM + 1)), with correlation analyses via the *cor.test* function (R). Supplementary tumor/normal datasets were acquired from the *Gene Expression Omnibus* (*GEO;*https://www.ncbi.nlm.nih.gov/gds) for cross-pathological validation.

### 2.7 Gene mutational landscape analysis

**Figure 2:**
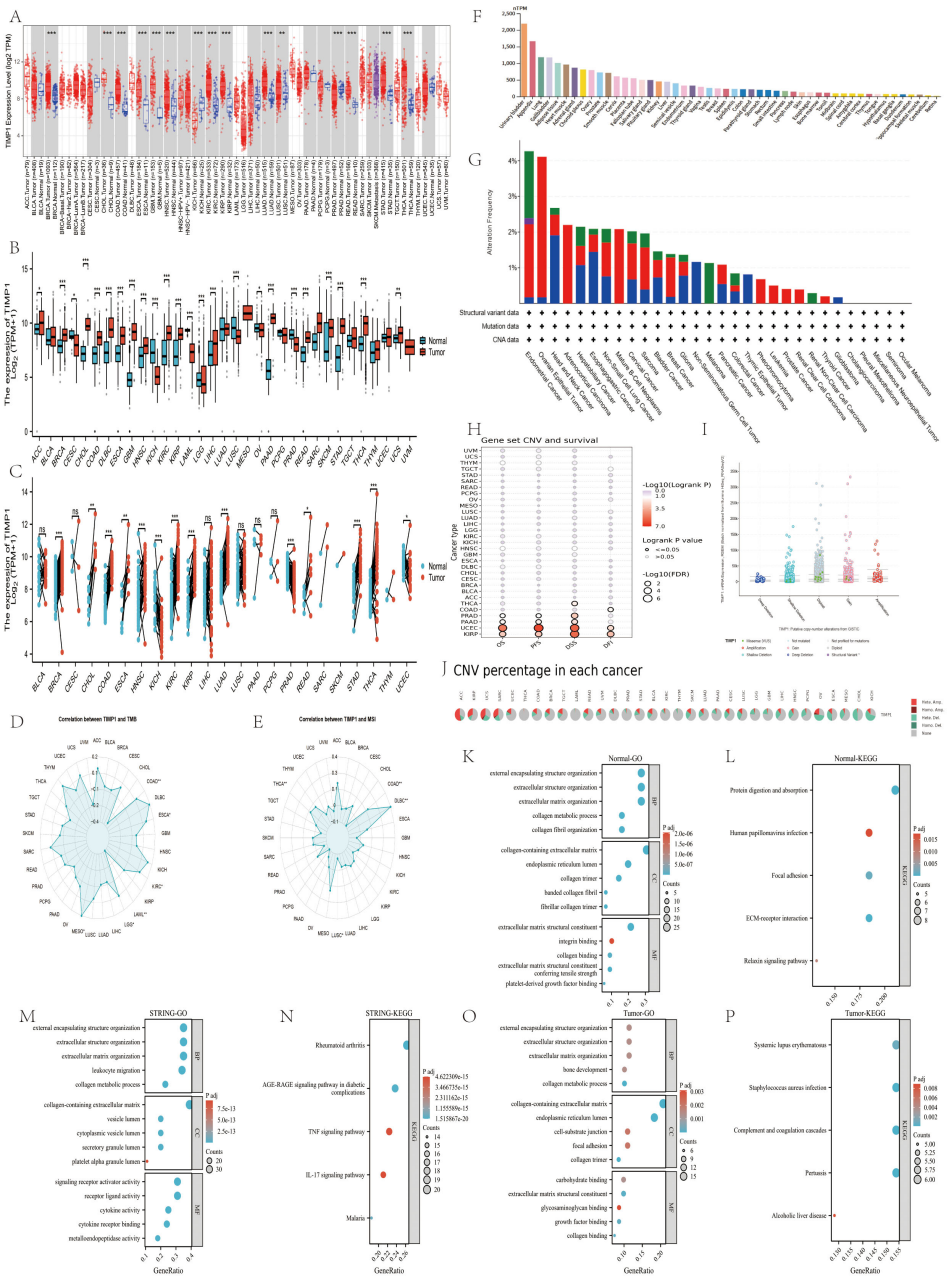
Pan-cancer landscape of TIMP1: expression, genomic alterations, and associations with TMB/MSI. **(A-C)** Pan-cancer TIMP1 expression across TCGA+GTEx; **(D, E)** correlations with TMB/MSI; **(F)** expression in normal tissues; **(G–J)** mutation and CNV landscape from *cBioPortal*. Panels **(K–P)** were consolidated as a single GO/KEGG analysis based tumor/normal tissue on co-expression (FDR<0.05). (**Figure 5**: Enhanced versions of **Figure 2J**).

Genomic alterations were analyzed via *cBioPortal (*http://www.cbioportal.org*)* using data from 10,960 tumor samples (10,528 pan-cancer patients) from *UCSC* Xena, *International Cancer Genome Consortium (ICGC;*https://www.icgc-argo.org*)*, and *TCGA Pan-Cancer Atlas* (log2(TPM + 1)). TIMP1 mutational landscape (mutation spectra, copy number variations [CNVs], prevalence) was characterized via the “Cancer Types Summary” module; somatic mutations were retrieved from *GDC*. Panels for mutation frequency, putative *GISTIC* CNA categories, and survival by CNA group (**Figures 2G–J**) were exported directly from *cBioPortal* (Cancer Types Summary/OncoPrint/Survival modules) using default portal settings. Reported p-values/HRs are portal-provided and treated as nominal (exploratory), without additional in-house multiple-testing harmonization.

### 2.8 Immune cell infiltration analysis

**Figure 3:**
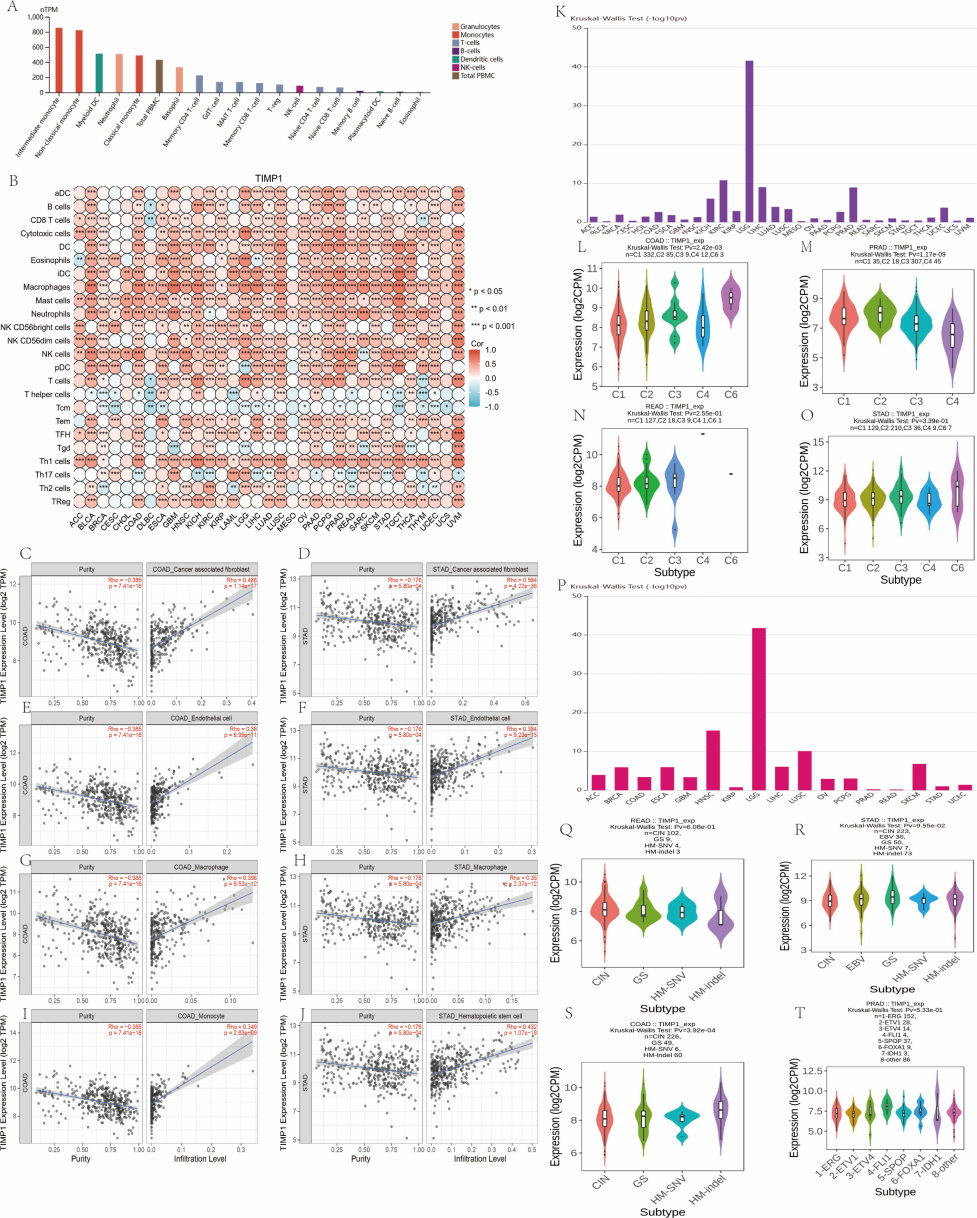
TIMP1 aligns with stromal–myeloid infiltration and the C6 immune subtype (COAD/STAD focus). **(A)** Expression in immune cell type. **(B)** Correlation analysis between TIMP1 and immune infiltration matrix data in pan – cancer. **(C, D)** Association between TIMP1 expression levels and fibroblast infiltration in COAD and STAD. **(E, F)** Correlation of TIMP1 expression with endothelial cell infiltration levels in COAD and STAD. **(G, H)** Correlation of TIMP1 expression with macrophage infiltration levels in COAD and STAD. **(I)** Correlation of TIMP1 expression with monocyte infiltration levels in COAD. **(J)** Correlation of TIMP1 expression with hematopoietic stem cell infiltration levels in STAD. **(K)** Associations between TIMP1 expression and immune subtypes across human cancers. **(L-O)** Expression of TIMP1 in the immune subtypes of COAD, PRAD, READ and STAD. **(P)** Associations between TIMP1 expression and molecular subtypes across human cancers. **(Q-T)** Expression of TIMP1 in the molecular subtypes of READ, STAD, COAD and PRAD. C1 (wound healing), C2 (IFN-gamma dominant), C3 (inflammatory), C4 (lymphocyte depleted), C5 (immunologically quiet) and C6 (TGF-b dominant).

Immune cell infiltration was quantified using *XCELL* (11); composition analyses were performed via *TIMER2 (*http://timer.cistrome.org/*)* (12) with focus on TIMP1 correlation. Immune cell distribution across TIMP1-stratified subtypes was visualized via heatmaps/boxplots (*ggplot2*, R). For pan-cancer correlations between TIMP1 and immune/stromal infiltration (**Figure 3B**), and fibroblast infiltration analyses in COAD/STAD (**Figures 3C, D**), we used *TIMER2* portal modules (Gene–Immune/Association) with the portal’s deconvolution backends. Outputs (correlation coefficients, nominal p-values) were exported from the portal and interpreted as exploratory; no additional in-house FDR harmonization was applied across cancer types.

No cell lines were involved in this analysis, and the cell lines referenced in the manuscript are exclusively associated with the experimental validation assays. Immune-cell infiltration analysis was performed using data of *TCGA* and *GEO* through computational tools. (#Data were from *XCELL*).

### 2.9 Methylation and mutation burden analysis​

TIMP1 promoter methylation was evaluated via *UALCAN (*https://ualcan.path.uab.edu/*)* (13), with comparative analysis via *GSCA (*https://guolab.wchscu.cn/GSCA*)* (14) to assess methylation-transcription associations (*Spearman’s analysis*, R). Relationships between TIMP1 and Tumor Mutational Burden (TMB) (15)/Microsatellite Instability (MSI) (16) were analyzed using TCGA multi-level data (*TCGA plot package*, R).

### 2.10 Prognostic analysis

TIMP1 prognostic significance was assessed via univariate Cox models and Kaplan-Meier survival analyses (survival, *survminer packages*, R). Clinical endpoints (overall survival [OS], disease-specific survival [DSS], disease-free survival [DFS], progression-free interval [PFI]) were from TCGA. Survival distributions were compared via log-rank tests unless otherwise specified, results refer to univariable models; effect sizes (HR, 95% CI) are emphasized.

### 2.11 Functional network and pathway enrichment analysis

Pathway enrichment was performed using *Kyoto Encyclopedia of Genes and Genomes (KEGG)* (17), *Gene Ontology (GO;*http://www.geneontology.org*)*, and the *clusterProfiler package* (R) (10) to identify TIMP1-associated biological processes.​ All enrichment results reported in the main text were computed or re-derived locally using *clusterProfiler* with BH-FDR control (q<0.05); figures initially screened via portals were validated in-house under the same FDR framework before reporting.

### 2.12 Drug sensitivity analysis​

TIMP1 chemotherapeutic sensitivity associations were analyzed using data from *GDSC* (https://www.cancerrxgene.org/) *(*18), *CTRP* (https://portals.broadinstitute.org/ctrp.v2.1) and *GSCA* (14). Drug–gene correlation screens were confirmed in-house under a unified Benjamini–Hochberg FDR procedure across agents within each dataset (q<0.05). Where portal modules were used for initial screening/visualization, final results shown in the manuscript reflect FDR-confirmed associations.

### 2.13 Differential expression analysis across clinical stages

TIMP1 differential expression across cancer clinical stages was analyzed via *ANOVA*, *Kruskal-Wallis tests*, *GEPIA2* (19) and *TISIDB* (20). Stage-/subtype-stratified comparisons shown in the manuscript were recomputed/validated in-house (*ANOVA* or *kruskal-wallis* + *Post hoc* comparison), and BH-FDR correction was performed on multiple comparisons (q<0.05); The portal chart is only for visual reference and should not be used as a basis for statistical conclusions.

### 2.14 ROC curve analysis

TIMP1 diagnostic performance was evaluated via ROC curves (*pROC package*, R) (21). Area Under the Curve (AUC) was calculated to quantify tumor/normal tissue discrimination.

### 2.15 ScRNA-seq data analysis

COAD scRNA-seq data (GSE146771) (22) and STAD scRNA-seq data (GSE134520) (23) were visualized via *TISCH* (https://tisch.compbio.cn/home/). Raw UMI counts were normalized (10,000 transcripts/cell) via *Seurat’s “NormalizeData”*; quality control, clustering, and annotation were performed via *MAESTRO*. While UMAP/annotation and other visualizations referred to TISCH. Quantitative analyses (differences, pathway enrichment/scoring, correlation) were all completed or reviewed in the local process before being reported in the main text.

### 2.16 Cardiovascular transcriptomic and network analyses​

TIMP1 in cardiovascular pathophysiology was analyzed using 2 GEO datasets (including GSE100927 and GSE5406) (24, 25). Differential expression was assessed via *limma* (R), with visualization via boxplots/ROC curves (*pROC*). *GSEA (MSigDB hallmarks)* and GO/KEGG enrichment *(clusterProfiler)* characterized TIMP1 associated networks.​ Enrichment analyses applied BH-FDR control where applicable (|logFC|≥1, adj.p<0.05). As this section focused on TIMP1-related network analyses rather than global differential expression, no DEG datasets were used.

### 2.17 Gene set enrichment analysis

GSEA was conducted using the *clusterProfiler package* (v4.4.4) (10) in R. The MSigDB c2.cp.all.v2022.1.Hs.symbols.gmt gene set (26) was used. Gene annotation and ID conversion were performed with *org.Hs.eg.db* and *msigdbr packages*. Enrichment results were visualized using bar plots, enrichment maps, and hierarchical clustering. For local differential and enrichment analyses, BH-FDR (q<0.05) was applied as implemented in *limma/clusterProfiler*.

### 2.18 Subcellular localization and immunohistochemical analysis

To validate our findings at the protein level, we accessed subcellular localization and immunohistochemical (IHC) staining data from the *Human Protein Atlas* (*HPA;*https://www.proteinatlas.org/). The *HPA* provides extensive IHC results for various tissues, including cancerous and normal tissues, using anti-TIMP1 antibodies. We utilized these publicly available IHC images and staining intensities to assess TIMP1 protein expression in different tumor types.

### 2.19 Western blotting

To explore TIMP1 expression and its role in gastric/colon cancer malignancy. Western Blotting (WB) (27) was performed to quantify TIMP1 protein levels. WB aimed to quantify TIMP1 in cancer vs. normal epithelial cells—cells were cultured under recommended conditions and total protein was isolated from cell panels (gastric cancer: AGS, MKN45, SGC7901 vs. normal GES-1; colon cancer: HCT116, SW480, LoVo vs. normal FHC), lysates prepared in RIPA buffer with protease/phosphatase inhibitors were cleared and quantified (BCA).

Equal protein (20–30 µg/lane) was resolved by 10–12% SDS-PAGE, transferred to PVDF, blocked (5% milk/TBST), and probed with anti-TIMP1 and GAPDH/β-actin (loading control). Signals were detected by ECL and densitometry was performed in ImageJ; TIMP1 intensities were normalized to the loading control and expressed relative to the matched normal line (GES-1 or FHC = 1.0). Unless stated otherwise, n=3 biological replicates; statistics used two-sided t-tests (pairwise) or one-way *ANOVA* with *Tukey post-hoc* for multi-group comparisons, applying BH-FDR when multiple testing occurred (q<0.05).

### 2.20 Transwell assays analysis

Transwell assays evaluated cancer cell migration and TIMP1’s impact: cells were grouped into empty vector control, TIMP1 overexpression (OE), and knockdown (KD); migration (uncoated inserts) was assessed using 24-well Transwells with 8 µm pores. Cells were serum-starved (12–16 h), seeded in serum-free medium into the upper chamber (typical: 5×10^4^/insert for migration), with 10% FBS medium in the lower chamber as chemoattractant. After incubation (migration 16–24 h; optimized per line), non-migrated cells were removed from the upper surface; membranes were fixed (4% PFA), stained with 0.1% crystal violet, and imaged at ~200×.

For each insert, five random non-overlapping fields were counted (ImageJ or blinded manual counting). Each condition used triplicate inserts and ≥3 independent experiments. Group differences among Control/OE/KD were analyzed by one-way *ANOVA* with *Tukey post-hoc*; pairwise by two-sided t-tests. Where multiple comparisons were performed within a figure/panel, BH-FDR adjustment was applied (q<0.05).

### 2.21 Statistical analysis​

Analyses were performed In *R 4.0.3 (RStudio)*. In-house computations uniformly applied Benjamini-hochberg FDR control where applicable (DEGs: logFC ≥1 and FDR<0.05; enrichment/pathway score: q<0.05; staging/typing comparison related to drug sensitivity: as described, q<0.05). Unless otherwise specified, these portal results are presented in nominal p and are only used as hypothesis-generating. Throughout the text, we emphasize the effect size (logFC, ρ, HR, 95%CI, AUC) and cross-dataset consistency.

## 3 Result

### 3.1 Dataset processing and differential gene expression analysis

**Figure 4:**
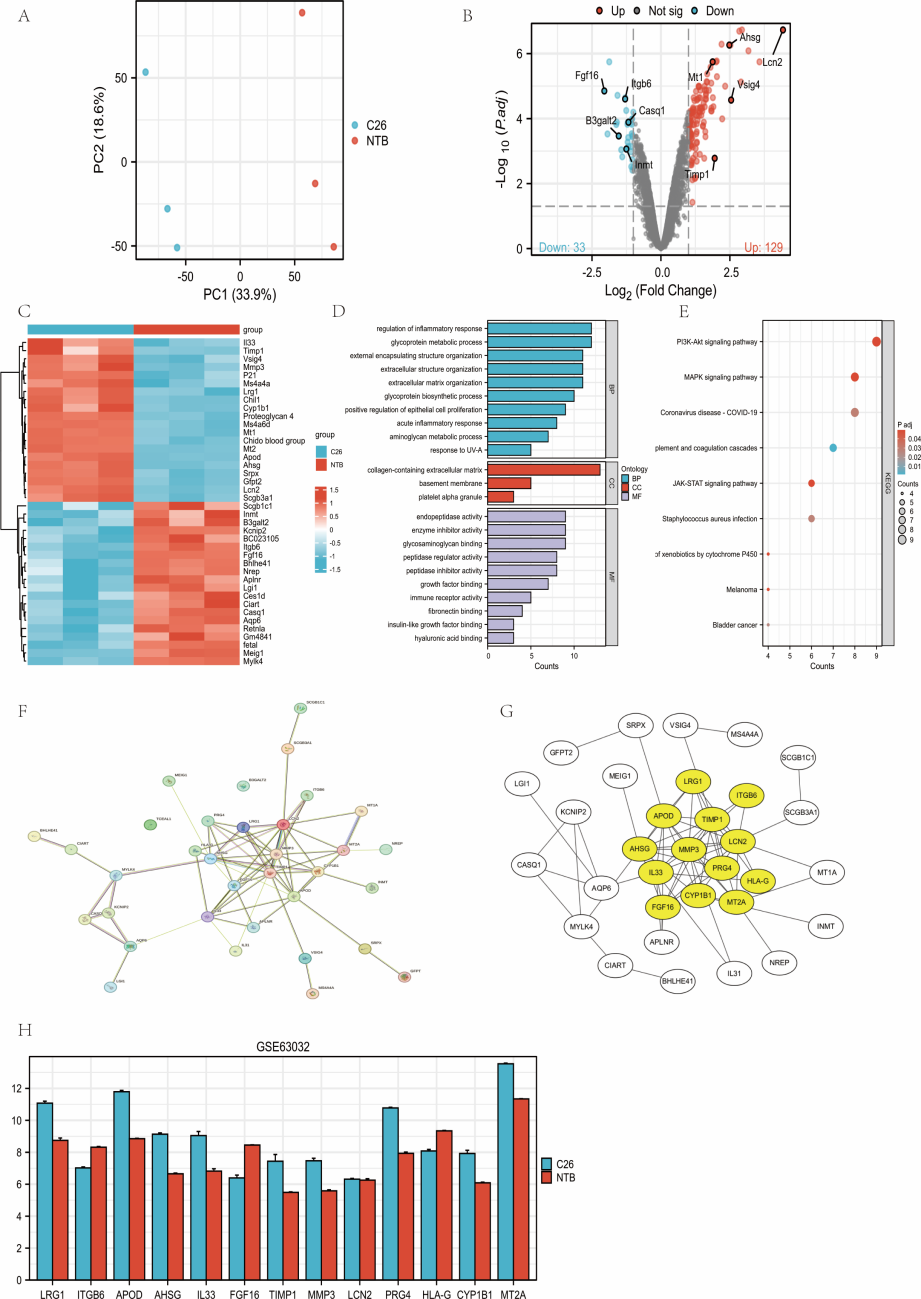
Differential expression and network prioritization in GSE63032 heart tissue identify TIMP1 as a hub. **(A)** Principal component analysis (PCA) illustrating the distribution of expression values across all samples. **(B)** Volcano plot of DEGs (*limma*; |logFC|≥1, FDR<0.05). **(C)** Heatmap depicting the top 20 upregulated and 20 downregulated DEGs. **(D, E)** GO/KEGG enrichment of DEGs(Top terms; FDR<0.05). **(F)** Protein-protein interaction (PPI) network of DEGs constructed via the *STRING* database. **(G)** The most significant module derived from the PPI network using *MCODE (Cytoscape)*. **(H)** Expression changes of the top 13 DEGs between C26 and non-tumor bearing (NTB) samples based on microarray dataset GSE63032. C26, colon cancer-bearing mice; NTB, non-tumor-bearing mice. (**Table 1**. The 162 DEGs).

In GSE63032 (heart; C26 = 3, NTB = 3), RMA normalization and batch correction yielded clear group separation by PCA(PC1 = 33.9%, PC2 = 18.6%; **Figure 4A**). Using *limma*(|logFC|≥1, FDR<0.05), we identified 162 DEGs (up 129/down 33; **Figure 4B**). Representative changes included Timp1 (logFC=1.95, FDR = 1.66E-03), Lcn2 (logFC=4.43, FDR = 1.87E-07) and Fgf16 (logFC=-2.05, FDR = 1.41E-05). The top 20 DEGs are shown in the heatmap (**Figure 4C**).

### 3.2 Functional enrichment analysis of DEGs

GO/KEGG (*clusterProfiler*) highlighted ECM/collagen organization (GO-BP/CC/MF) and PI3K-Akt, MAPK, ECM–receptor interaction (KEGG) as significantly enriched (all FDR<0.05; **Figures 4D, E**). (This analysis utilized the full dataset of 162 DEGs identified in Section 2.1).

### 3.3 Protein-protein interaction network construction and module identification

*STRING* built a PPI network from 162 DEGs (34 nodes;**Figure 4F**). *MCODE* extracted a 13-gene module (**Figure 4G**), with Timp1 ranking Top-k by degree/MCODE score and being upregulated in C26 myocardium compare to NTB (**Figure 4H**), thereby nominating TIMP1 for downstream analyses. (This analysis utilized the full dataset of 162 DEGs identified in Section 2.1).

### 3.4 Expression profiling across cancers

Across cancers, TIMP1 showed widespread upregulation in *TIMER2* and *TCGA–GTEx* analyses (**Figures 2A–C**), with consistent elevation in COAD (n=457), READ (n=166), STAD (n=415), BRCA (n=1093), ESCA (n=184), HNSC (n=520), KIRC (n=533), KIRP (n=290), LUAD (n=515), and LUSC (n=501), while KICH (n=66) remained the only tumor type with persistent downregulation.

TIMP1 expression also correlated with TMB and MSI, particularly in COAD, KIRC, MESO, and DLBC (**Figures 2D, E**), suggesting potential links to mutational burden and microsatellite variation. Normal-tissue profiling indicated clear baseline heterogeneity, with the highest expression in the bladder and appendix and the lowest in the cerebellum and retina (**Figure 2F**). These observations support a broadly increased and context-dependent expression pattern of TIMP1 across malignancies.

### 3.5 Mutation and copy number variation analysis

cBioPortal summarizes TIMP1 alterations in ≥25 cancer types, predominantly CNV (gain/deletion) (**Figure 2G**). In UCEC/KIRP, CNA groups show survival differences (OS/PFS/DSS/DFI; **Figure 2H**). GISTIC-based categories associate with higher TIMP1 mRNA in Gain/Diploid groups (**Figures 2I–J**). Digestive cancers (COAD/READ/STAD) are enriched for heterozygous CNAs (**Figures 2I–J**). These genomic features align with the expression patterns above while remaining correlative.

### 3.6 Functional enrichment analysis of TIMP1

Functional enrichment of TIMP1-coexpressed genes identified extracellular matrix organization, collagen-associated pathways, and ER-lumen or secretory processes as the predominant functional signatures across GO, KEGG, and *STRING* analyses (**Figures 2K–P**). In normal tissues, additional enrichment was observed for ECM structural components and protein digestion or absorption, while *STRING* analysis highlighted immune-inflammatory ECM signaling. Together, these convergent results indicate that TIMP1 is primarily embedded within an ECM-centered functional context and participates in collagen remodeling and matrix-associated regulatory processes across tumor types.

### 3.7 Prognostic and diagnostic value analysis

Multivariate Cox regression analyses, along with representative Kaplan-Meier survival curves, reveal that elevated levels of TIMP1 are correlated with poorer overall survival (OS), disease-specific survival (DSS), progression-free interval (PFI), and disease-free interval (DFI) across various malignancies.

This trend is notably consistent in colorectal cancers (COAD/READ/STAD), as well as in kidney renal clear cell carcinoma (KIRC), uveal melanoma (UVM), and kidney renal papillary cell carcinoma (KIRP). Conversely, adrenocortical carcinoma (ACC) and skin cutaneous melanoma (SKCM) exhibit protective associations (hazard ratio < 1) (**Figures 6A–F**; nominal p < 0.05).

**Figure 5:**
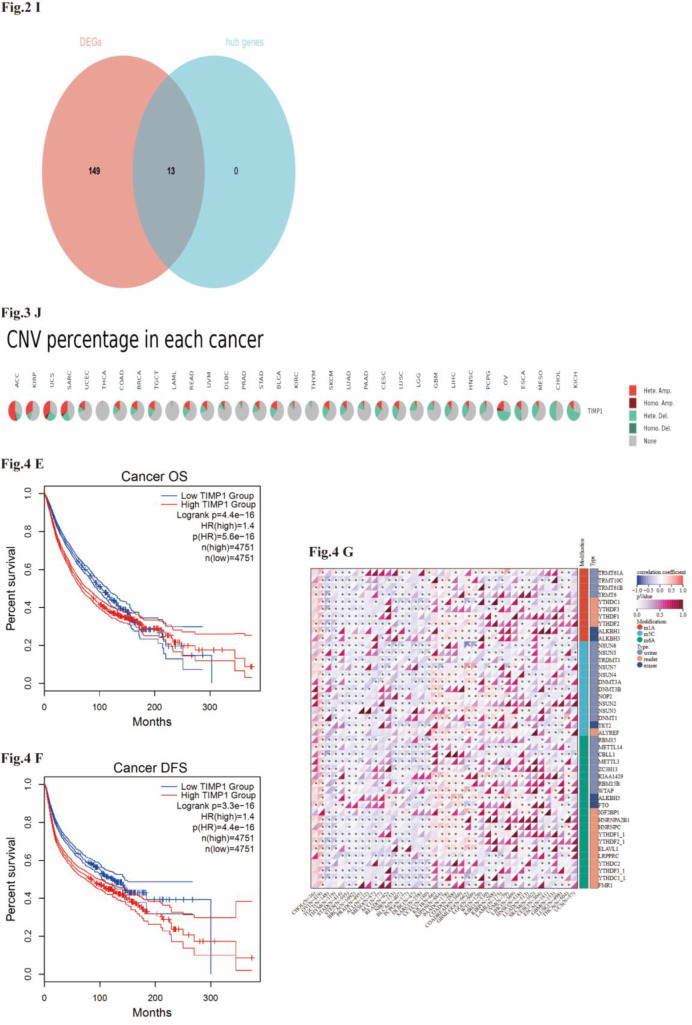
Enhanced versions of **Figure 2J**, **Figures 6E–G**, and the supplementary Venn diagram of DEGs and hub genes.

**Figure 6:**
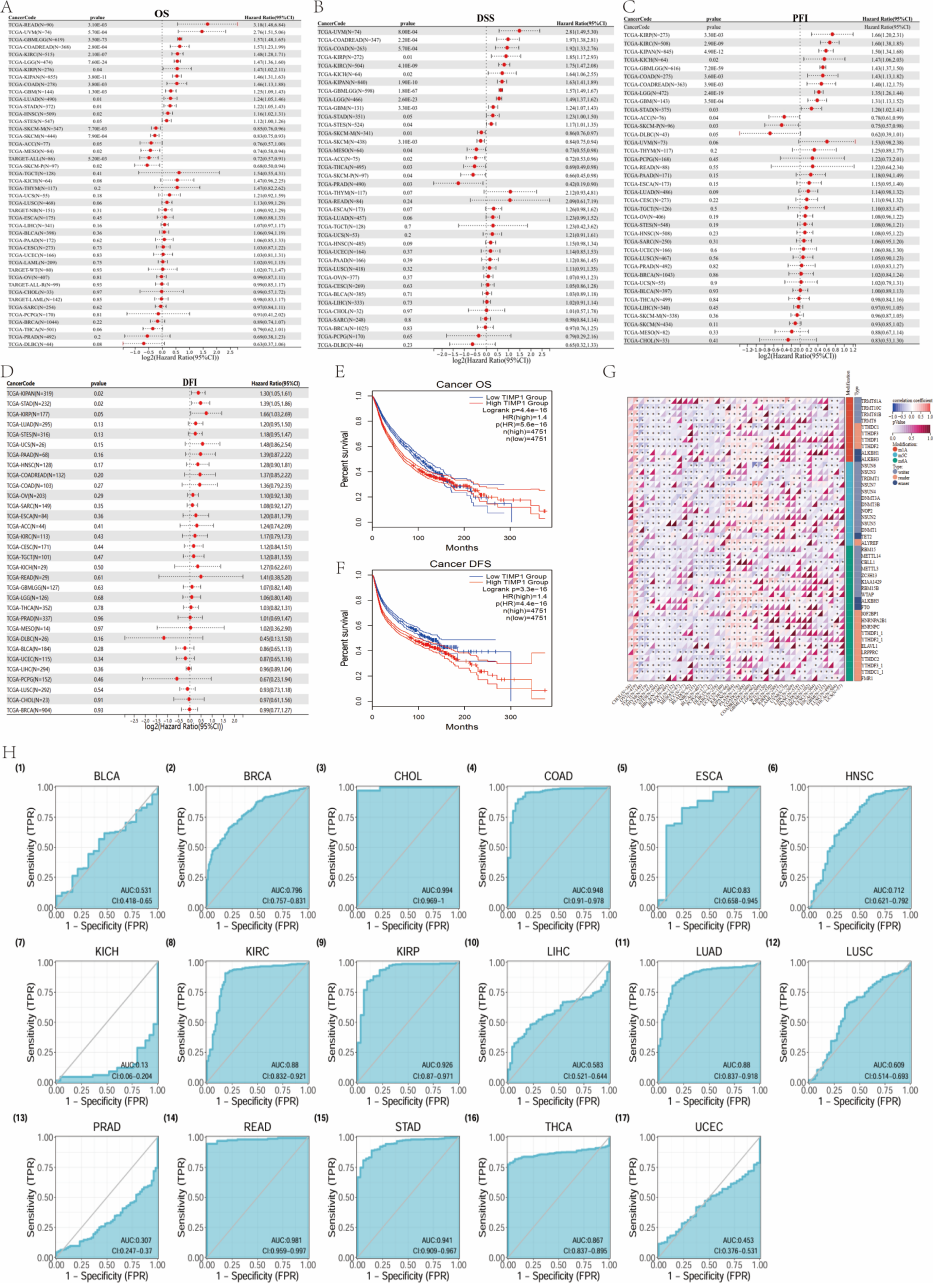
Prognostic value of TIMP1 expression across multiple cancer types in TCGA pan-cancer data. **(A–D)** Univariate Cox forests for OS/DSS/PFI/DFI; **(E, F)** representative KM curves (DFS: Disease-Free Survival); **(G)** correlations between TIMP1 and m1A/m5C/m6A regulators; **(H)** multi-cancer diagnostic ROC panels *(The main text only retains cancer types with AUC>0.85.*) The P-values provided by Portal are interpreted exploratory. The figure shows effect sizes such as HR, 95%CI, and AUC. (**Figure 5** Enhanced versions of **Figures 4E–G**).

The diagnostic receiver operating characteristic (ROC) panels demonstrate an area under the curve (AUC) of 0.90 or greater for cholangiocarcinoma (CHOL), COAD, READ, STAD, and KIRP (**Figure 6H**, ROC matrix). Collectively, tumors of the digestive system display the most pronounced adverse prognostic implications, characterized by high diagnostic accuracy among the specified cancer types. These observations underscore the correlation and highlight the significance of effect sizes and consistency across different panels.

### 3.8 Association analysis between TIMP1 and mRNA modifications

Across cancers, TIMP1 shows significant correlations with m1A/m5C/m6A regulatory factors, with consistent signals in COAD, STAD, COADREAD (**Figure 6G**; nominal p<0.05). Directions and strengths are reported in the panel; these results are hypothesis-generating and require experimental validation to address causality.

### 3.9 Methylation status analysis

**Figure 7:**
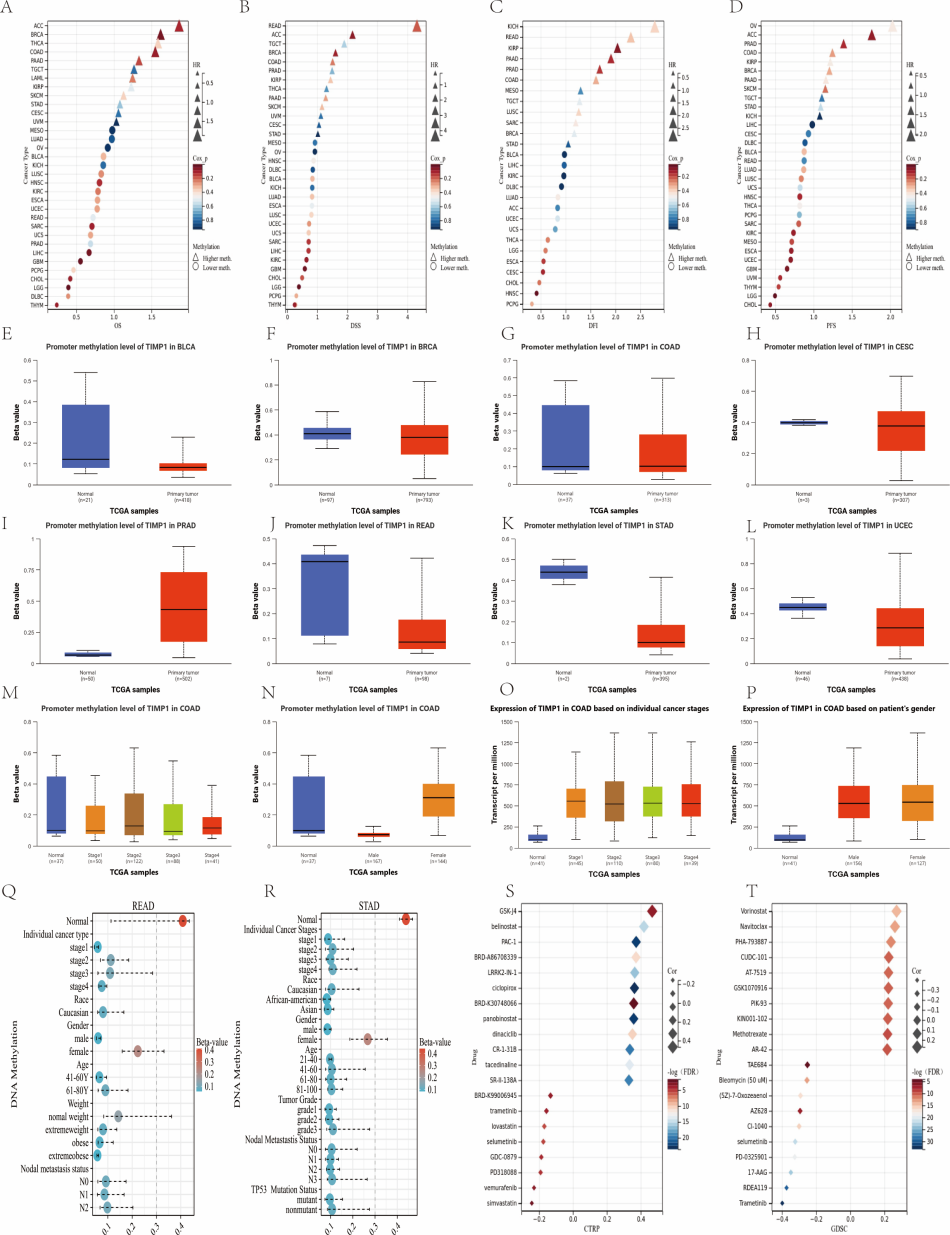
TIMP1 promoter methylation patterns and pharmacologic response across cancers. **(A–D)** Survival associations stratified by TIMP1 promoter methylation (OS/DSS/DFI/PFS). **(E–L)** Promoter methylation status of TIMP1 across BLCA, BRCA, COAD, CESC, PRAD, READ, STAD, and UCEC cohorts. **(M)** Stage-stratified promoter methylation of TIMP1 in COAD. **(N)** Gender-specific promoter methylation of TIMP1 in COAD. **(O)** TIMP1 expression in COAD grouped by cancer stage. **(P)** TIMP1 expression in COAD categorized by patient gender. **(Q)** Schematic representation of TIMP1 methylation-expression patterns across diverse READ sample types. **(R)** Schematic illustration of TIMP1 methylation-expression profiles in various STAD sample types. **(S, T)** Correlation between TIMP1 expression and CTRP/GDSC drug sensitivity (top 20 agents) in pan-cancer contexts.

Across cancers, survival panels (**Figures 7A–D**) show methylation–outcome associations that vary by tumor type; representative patterns include higher methylation aligning with worse OS/DSS in READ/ACC/BRCA, whereas the opposite trend is observed in GBM/THYM. We focus on effect sizes (HR, 95%CI) as displayed in the figure.

Tumor-normal comparisons (**Figures 7E–L**) indicate lower promoter methylation in tumors for COAD/READ/STAD/UCEC While BRCA/CESC/PRAD are in the opposite direction; The details of the cancer types are shown in the caption of the figure. In COAD, methylation decreases while expression increases across stages (**Figures 7M, O**), and female patients show slightly higher promoter methylation than males (**Figures 7N, P**). In READ/STAD, promoter methylation is consistently lower than normal across stages (**Figures 7Q–R**). Together, these panels outline stage and sex-associated methylation–expression patterns that warrant stratified analyses.

Summary. TIMP1 promoter methylation shows cancer-type–specific relationships with outcome, and inverse methylation–expression trends in colorectal/gastric contexts, highlighting a potential stratification axis.

### 3.10 Drug sensitivity correlation analysis

Across CTRP/GDSC, higher TIMP1 expression correlates with greater sensitivity to epigenetic modulators (e.g., HDAC inhibitors Belinostat/Vorinostat; FDR<0.05) and JMJD3/UTX inhibitors (GSK-J4; FDR<0.05), and was associated with reduced sensitivity of MAPK inhibitors (MEK/BRAF, such as Trametinib/Vemurafenib) and metabolic inhibitors (HMM-CoA reductase, such as Simvastatin) (FDR<0.05 **Figures 7S-T**). The results were classified by pharmacological category. The effect sizes and correction methods are shown in the notes to the figure.

### 3.11 Single-cell RNA sequencing analysis

**Figure 8:**
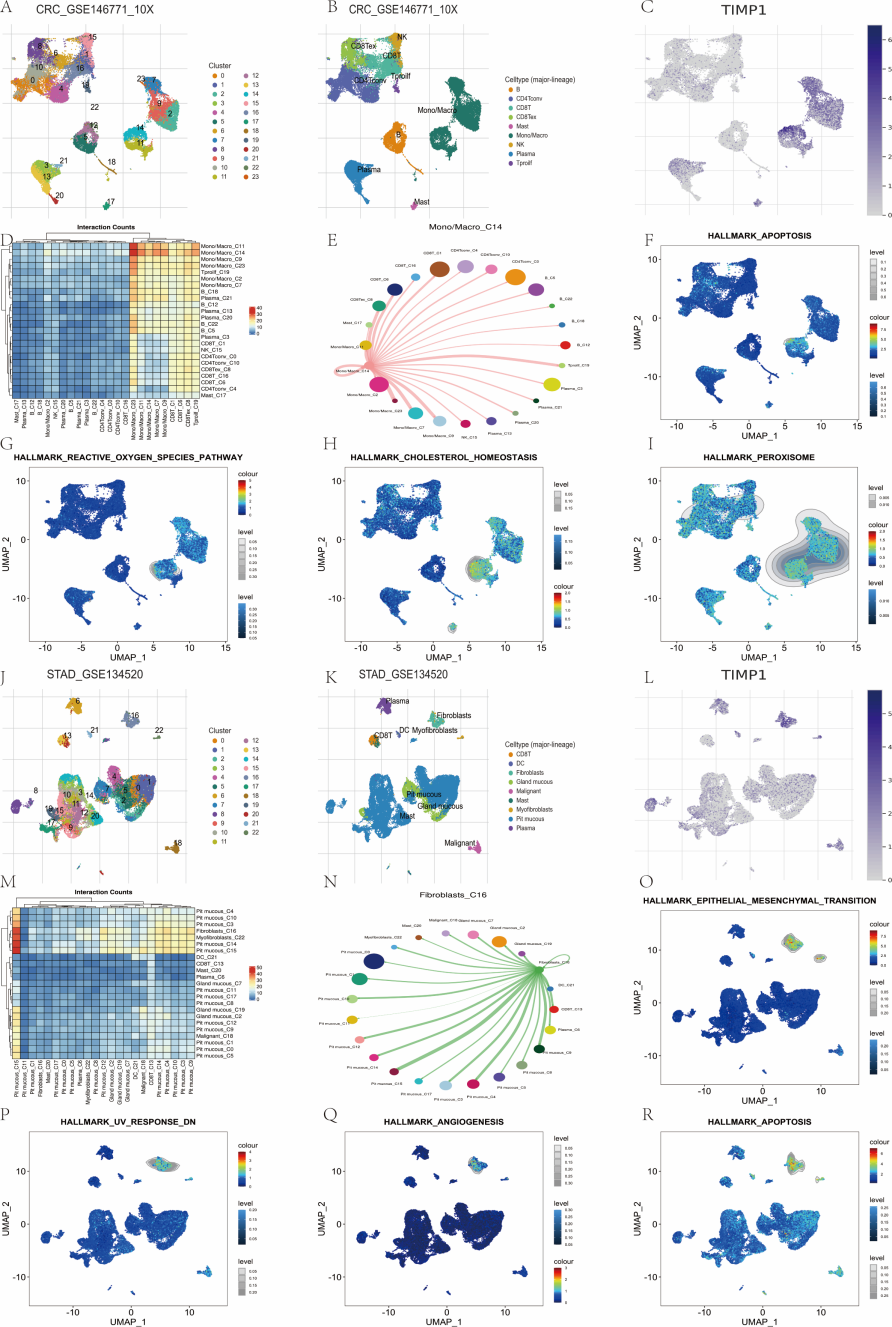
Single-cell distribution, pathway scores, and interaction context of TIMP1 in COAD and STAD. **(A)** Cellular clusters were identified using the CRC_GSE146771 dataset. **(B)** Cluster classification was performed based on predominant lineages within the dataset. **(C)** TIMP1 expression levels were evaluated across tissue-derived cell types isolated from the CRC_GSE146771 dataset. **(D)** Interaction frequencies among cellular clusters were quantified. **(E)** Cell-cell communication patterns between C14 Mono/Macro populations and other clusters were analyzed. **(F-I)** Single-cell level enrichment of the HALLMARK APOPTOSIS, REACTIVE OXYGEN SPECIES PATHWAY, CHOLESTEROL HOMEOSTASIS and PEROXISOME pathway was examined. **(J)** Cellular clusters were identified using the STAD_GSE134520 dataset. **(K)** Cluster classification was performed based on predominant lineages within the dataset. **(L)** TIMP1 expression levels were evaluated across tissue-derived cell types isolated from the STAD_GSE134520 dataset. **(M)** Interaction frequencies among cellular clusters were quantified. **(N)** Cell-cell communication patterns between C16 fibroblasts populations and other clusters were analyzed. **(O-R)** Single-cell level enrichment of the HALLMARK EPITHELAL MESENCHYMAL TRANSITION, UV RESPONSE DN, ANGIOGENESIS and APOPTOSIS pathway was examined.

In the context of COAD (GSE146771), the application of unsupervised clustering techniques resulted in the identification of 24 distinct clusters spanning 9 lineages (**Figures 8A, B**). Notably, TIMP1 exhibited the highest expression levels in the Mono/Macro_C14 cluster, with a prevalence of 95.4% and log2FC of 2.2 (**Figure 8C**). Interaction analysis revealed a more pronounced connectivity with the Mono/Macro_C11 and Mono/Macro_C23 clusters (**Figures 8D, E**). Gene-set scoring analyses indicated significant positive correlations between TIMP1 expression and the scores for HALLMARK APOPTOSIS and REACTIVE OXYGEN SPECIES (**Figures 8F–I**). It is important to note that the conclusions drawn are correlational in nature, with effect sizes and statistical values detailed within the corresponding panels.

In the case of STAD (GSE134520), a total of 23 clusters were identified across 9 lineages (**Figures 8J–K**). The Fibroblasts_C16 cluster demonstrated the highest TIMP1 expression, with a percentage of 83.7% and a log2FC of 1.75 (**Figure 8L**), alongside stronger associations with the Pit mucous_C15 and Pit mucous_C4 clusters (**Figures 8M–N**). TIMP1 levels exhibited positive correlations with scores for HALLMARK EPITHELIAL MESENCHYMAL TRANSITION and ANGIOGENESIS (**Figures 8O–R**).

A comparative analysis across cohorts underscores the specificity of cell types, highlighting the presence of Mono/Macro_C14 (myeloid) in COAD as opposed to Fibroblasts_C16 (stromal) in STAD. Both cohorts demonstrated positive correlations with APOPTOSIS scores; however, COAD exhibited a tendency towards reactive oxygen species (ROS)-related metabolic stress, whereas STAD was aligned with epithelial-mesenchymal transition (EMT) and angiogenesis-related pathways. These observations suggest a TIMP1–tumor microenvironment (TME) phenotype that is contingent upon cell-type specificity (correlative).

In summary, TIMP1 is characterized by elevated expression levels that are specific to the myeloid compartment in COAD and the fibroblast compartment in STAD, which correspond with variations in pathway scores; however, the mechanistic implications of these findings necessitate further functional validation.

### 3.12 Immune infiltration and subtype association analysis

**Figure 3A** shows TIMP1 expression across immune cell subtypes: significantly elevated in intermediate/non-classical monocytes, and markedly lower in naïve B cells and eosinophils. **Figure 3B** (Spearman correlation analysis, pan-cancer datasets) reveals that in COAD/STAD, TIMP1 is predominantly positively correlated with immune cells (dendritic cells, macrophages, mast cells) and negatively correlated with Th17 cells and T helper cells; overall, TIMP1 has significant positive correlations with most immune cell types, supporting its pan-cancer immunomodulatory associations.

Across COAD and STAD, TIMP1 shows the strongest positive associations with cancer-associated fibroblasts (CAF), endothelial cells, and macrophages (ρ≈0.35–0.58; **Figures 3C–H**). Signals are directionally consistent across XCELL deconvolution backends. Associations with monocytes and hematopoietic stem cells are present but secondary (**Figures 3I, J**). By immune subtype, TIMP1 is higher in C6 (TGF-β-dominant) in both COAD and STAD (*Kruskal–Wallis*; **Figures 3K–O**). Molecular-subtype patterns are summarized in **Figures 3P–T**.

**Figure 3P** shows TIMP1’s associations with molecular subtypes: READ subtypes have consistent TIMP1 expression except for divergent CIN levels (**Figure 3Q**); STAD’s HM-indel subtype has diminished expression (**Figure 3R**), whereas COAD’s HM-indel has significantly elevated levels (**Figure 3S**); PRAD’s IDH1-mutant subtype has distinct TIMP1 expression (**Figure 3T**).

In summary, TIMP1 aligns with a stromal–myeloid–enriched pattern (CAF/endothelial/macrophage) and higher levels in the C6 immune subtype. Three guardrails: (i) concordance across deconvolution methods, (ii) replication in COAD and STAD, and (iii) emphasis on effect sizes with multiple-testing adjustment where applicable.

### 3.13 External dataset validation using GEO

**Figure 9:**
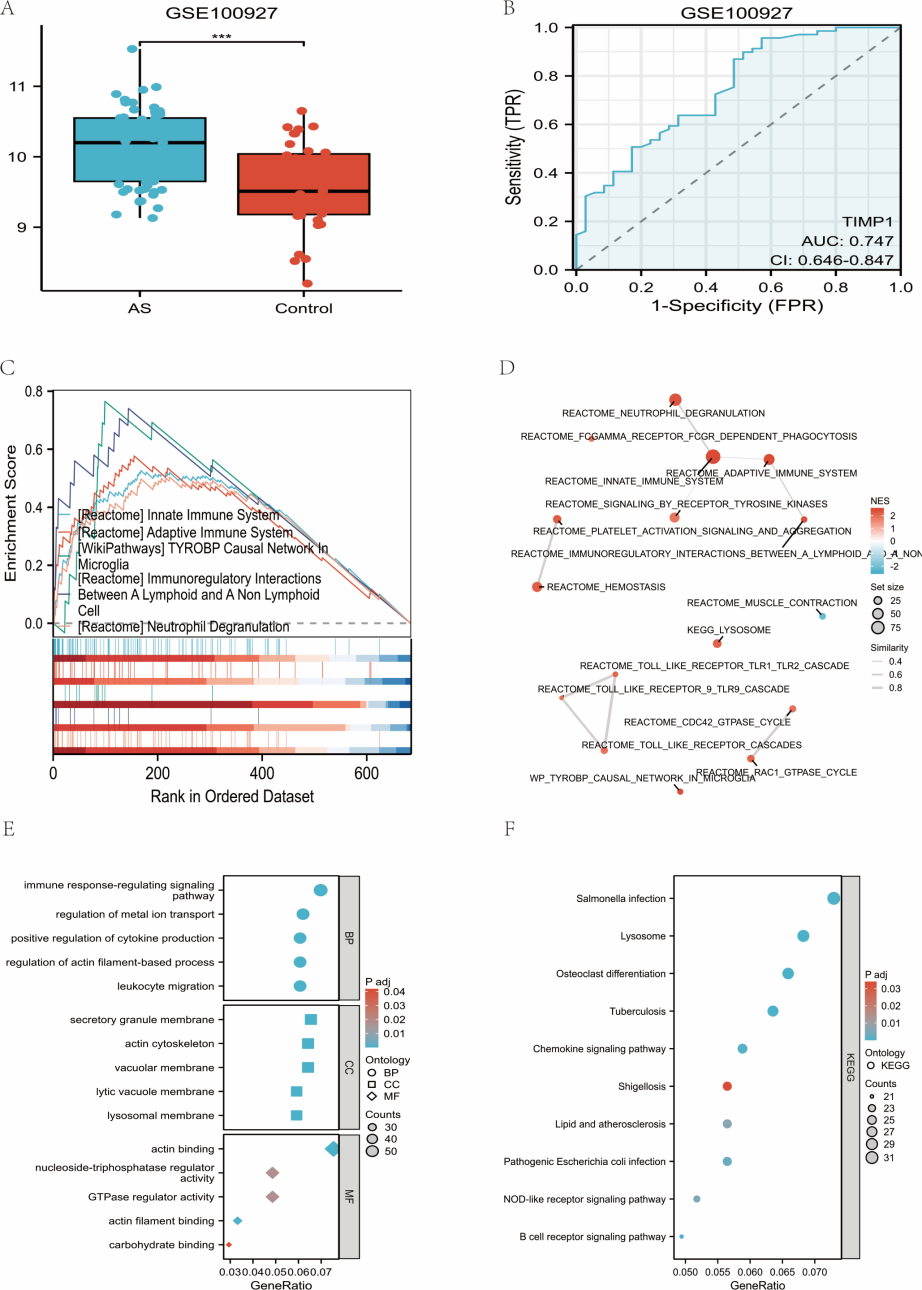
TIMP1 is elevated in atherosclerosis and aligns with immune/TLR and lipid–atherosclerosis pathways (GSE100927). **(A)** Comparative boxplot analysis of TIMP1 mRNA expression levels in atherosclerotic lesions (AS; dataset GSE100927) versus healthy arterial controls. **(B)** Receiver operating characteristic (ROC) curve analysis evaluating the diagnostic performance of TIMP1 in discriminating atherosclerotic lesions from normal tissues within the GSE100927 cohort. **(C)** Gene set enrichment analysis (GSEA) results ranked by normalized enrichment score (NES), highlighting the top 5 positively enriched pathways. **(D)** Enrichment map (EMAP) visualization of the 16 enriched pathways. **(E)** Gene Ontology (GO) analysis of TIMP1 correlated genes in the GSE100927 dataset. **(F)** KEGG pathway analysis of TIMP1 correlated genes in the GSE100927 dataset.

In the analysis of GSE100927 pertaining to atherosclerosis, it was observed that the levels of TIMP1 were significantly elevated in lesion samples compared to control samples, with a false discovery rate (FDR) of less than 0.001 (**Figure 9A**). The area under the curve (AUC) for distinguishing between cases and controls was determined to be 0.747 (**Figure 9B**).

Gene Set Enrichment Analysis (GSEA) revealed that the most notable enriched biological processes included innate and adaptive immune responses, interactions within the TYROBP network, immunoregulatory interactions, and the process of neutrophil degranulation, with these being the top five terms ranked by normalized enrichment score (NES) and an FDR of less than 0.05 (**Figure 9C**). Furthermore, the Enrichment Map (EMAP) method categorized the enriched terms into Toll-like receptor (TLR) signaling cascades, specifically TLR1–TLR2, TLR9, and a broader TLR cluster (**Figure 9D**).

Additionally, GO enrichment showed that TIMP1 related biological processes (BP) were mainly involved in immune response regulation, leukocyte migration, and cytokine production. Enriched cellular components (CC) included lysosomal and vacuolar membranes as well as the actin cytoskeleton, and enriched molecular functions (MF) included actin binding and GTPase regulator activity (**Figure 9E**). KEGG pathways further highlighted TIMP1 associated processes, including chemokine signaling, lysosome function, lipid and atherosclerosis pathways, and NOD like receptor signaling, all with FDR < 0.05 (**Figure 9F**).

The findings indicate that TIMP1 is significantly elevated in atherosclerotic tissues, providing moderate diagnostic utility with an AUC of 0.747. The enrichment analyses consistently align with pathways related to innate and adaptive immune responses, TLR-centered signaling modules, neutrophil activity, and lipid metabolism, thereby reinforcing the relevance of an inflammation-immune axis in the context of atherosclerosis.

### 3.14 External dataset validation Using GEO

**Figure 10:**
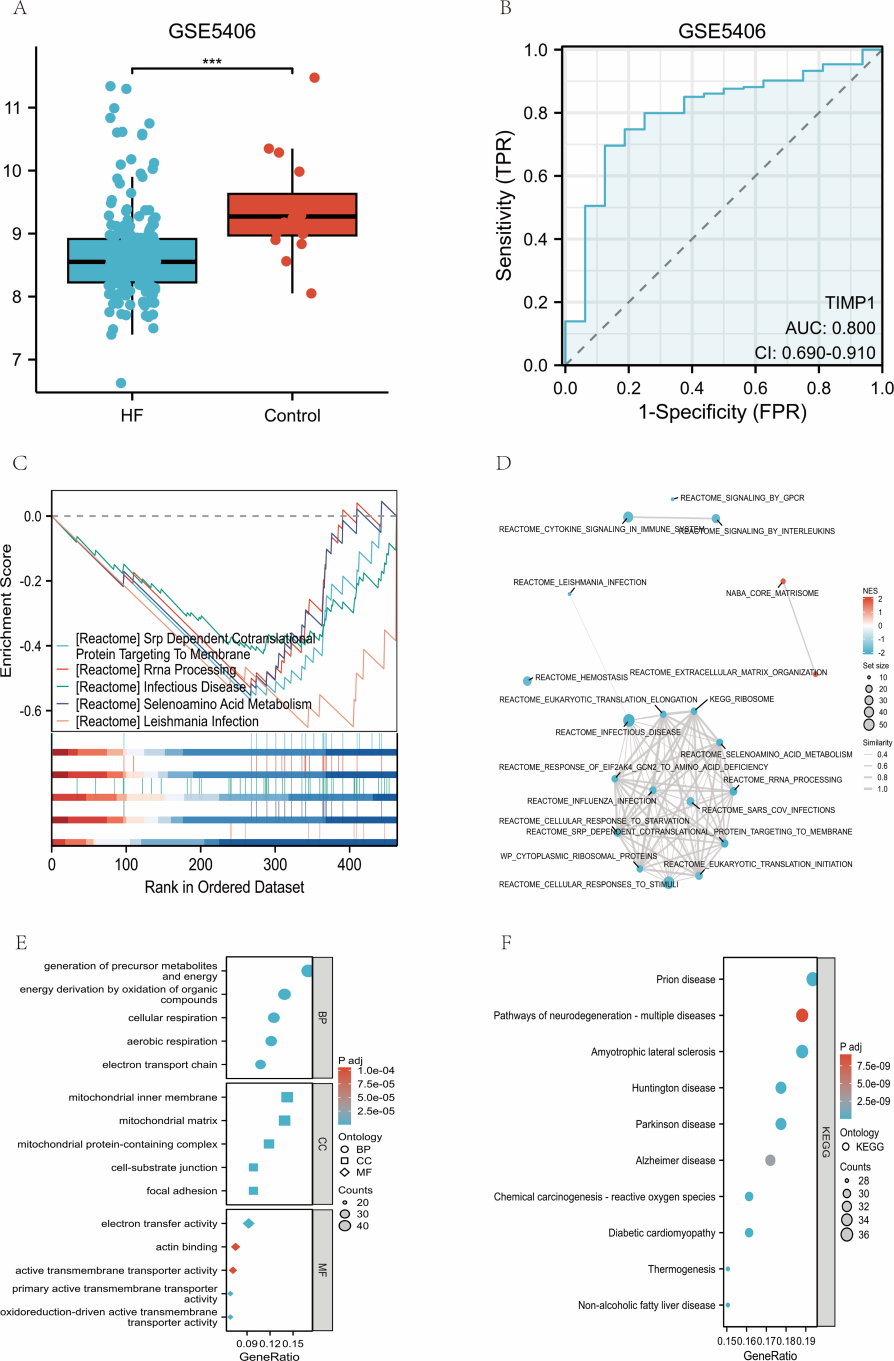
TIMP1 is reduced in heart failure and aligns with mitochondrial/energy-metabolism signatures (GSE5406). **(A)** Comparative boxplot analysis of TIMP1 mRNA expression levels in heart failing (HF; dataset GSE5406) versus healthy heart. **(B)** Receiver operating characteristic (ROC) curve analysis evaluating the diagnostic performance of TIMP1 in discriminating HF from normal tissues within the GSE5406 cohort. **(C)** Gene set enrichment analysis (GSEA) results ranked by normalized enrichment score (NES), highlighting the top 5 negatively enriched pathways. **(D)** Enrichment map (EMAP) visualization of the 20 enriched pathways. **(E)** Gene Ontology (GO) analysis of TIMP1 correlated genes in the GSE5406 dataset. **(F)** KEGG pathway analysis of TIMP1 correlated genes in the GSE5406 dataset.

In the dataset GSE5406 pertaining to heart failure (HF), it has been observed that the levels of TIMP1 are significantly diminished in the myocardial tissues of HF patients compared to control subjects (FDR<0.01; **Figure 10A**). The area under the curve (AUC) for distinguishing between cases and controls is recorded at 0.800 (**Figure 10B**).

Gene Set Enrichment Analysis (GSEA) of TIMP1-related genes in GSE5406 shows that the negatively enriched gene sets converge on pathways involved in mitochondrial oxidative phosphorylation, electron transport, translation, ribosomes, and signal recognition particle targeting, consistent with the specific NES-ranked pathways including Srp-dependent cotranslational protein targeting to membrane, rRNA processing, infectious disease pathways and selenoamino acid metabolism (FDR<0.05; **Figure 10C**). Furthermore, the Enrichment Map Analysis Project (EMAP) organizes these findings into two main clusters: translation/ribosome and stress-response modules (**Figure 10D**).

The results from Gene Ontology (GO) and Kyoto Encyclopedia of Genes and Genomes (KEGG) analyses are consistent, highlighting terms associated with energy metabolism and respiration (Biological Process, BP), components of the mitochondrial inner membrane and matrix (Cellular Component, CC), as well as electron transfer activity (Molecular Function, MF). Additionally, various disease pathways are noted, encompassing diabetic cardiomyopathy, reactive oxygen species (ROS)-related pathways, thermogenesis, and neurodegenerative disorders (FDR<0.05; **Figures 10E, F**).

The downregulation of TIMP1 in the context of HF is concomitant with alterations in mitochondrial energy metabolism and translation-related gene expression profiles.

### 3.15 Subcellular localization and IHC (COAD)

**Figure 11:**
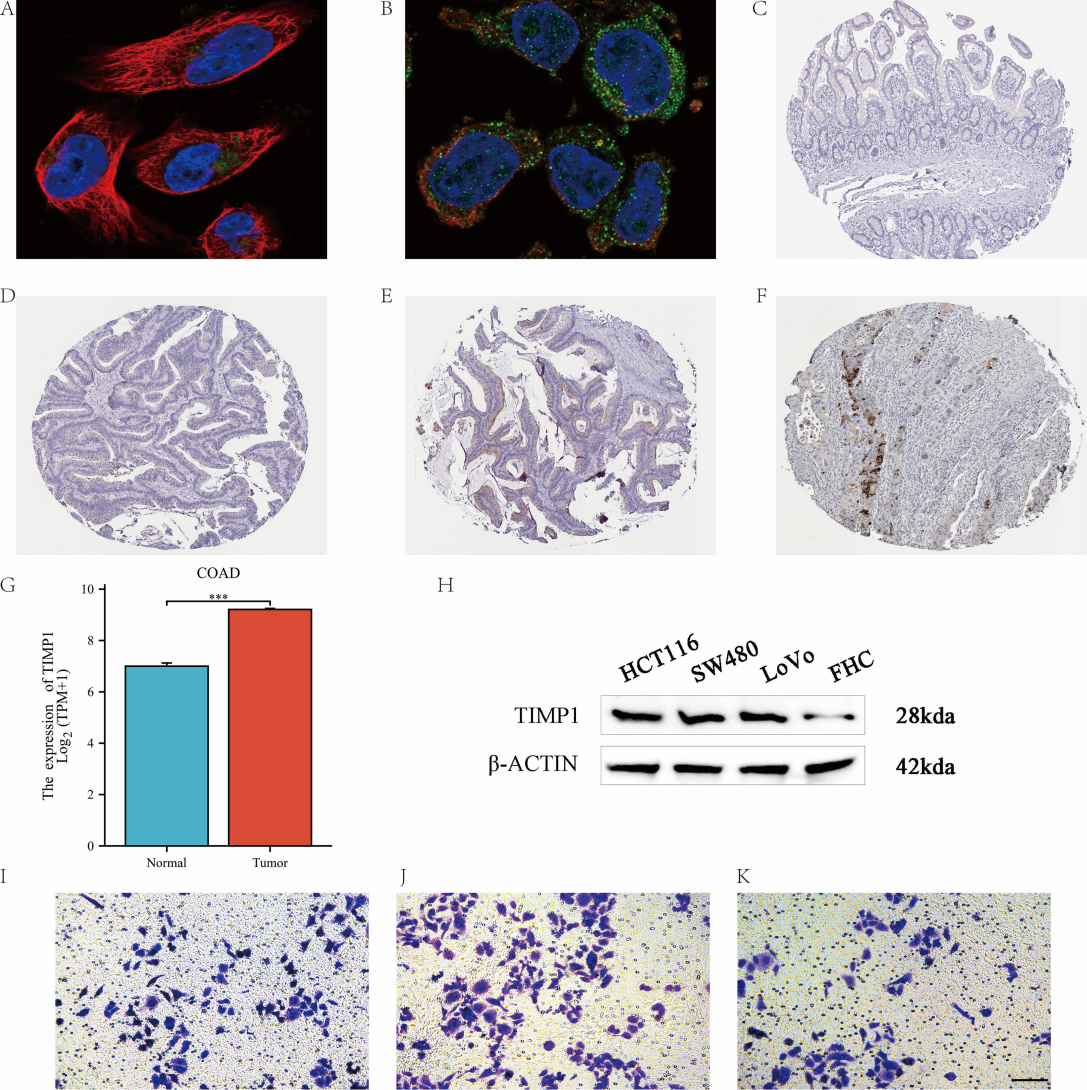
TIMP1 subcellular localization and colorectal validation (COAD): IHC, tissue RNA, Western blot, and Transwell assays. **(A, B)** Immunofluorescence in U-251MG and THP-1 shows perinuclear/Golgi and vesicular localization of TIMP1. **(C–F)** Colon IHC: representative normal vs colorectal adenocarcinoma (COAD) sections stained for TIMP1. **(C)** low/absent staining in normal colon mucosa; **(D-F)** increased staining in COAD tissue epithelium/stroma. **(G)** Tissue RNA expression: TIMP1 levels in COAD vs adjacent normal(TCGA). **(H)** Western blot: TIMP1 protein in FHC (normal) vs COAD cell lines (HCT116, SW480, LoVo) with GAPDH/β-actin loading controls; densitometry normalized to FHC = 1.0 (n=3 biologic replicates). **(I–K)** Transwell migration assays using the HCT116 colorectal cancer (COAD) cell line under empty vector control **(I)**, TIMP1 overexpression (**J**, OE) (), and knockdown (**K,** KD). Representative micrographs and quantification (mean ± SD, triplicate inserts; ≥3 independent experiments). Group comparisons by one-way *ANOVA* with *Tukey post-hoc*; when multiple pairwise tests were performed within a panel, BH–FDR correction was applied (q<0.05 considered significant). “OE” refers to TIMP1 overexpression, and “KD” refers to TIMP1 knockdown.

Localization of TIMP1 to the Golgi apparatus (**Figure 11A**) and cytoplasmic vesicles (**Figure 11B**) was detected by immunofluorescence. Subsequent immunohistochemical assessment further corroborated TIMP1 expression patterns within both normal and tumor tissues. Comparative analysis revealed minimal TIMP1 immunoreactivity in normal colonic specimens (**Figure 11C**), whereas markedly upregulated expression was demonstrated in colorectal cancer tissues (**Figures 11D–F**). Immunofluorescence showed perinuclear Golgi and vesicular localization (U-251MG, THP-1; **Figures 11A, B**), consistent with a secreted protein. IHC demonstrated low/absent staining in normal colon and increased staining in colorectal cancer (**Figures 11C–F**).

### 3.16 COAD validation: tissue RNA, WB, and Transwell migration

In colorectal adenocarcinoma (COAD) tissues, TIMP1 mRNA and protein levels were significantly elevated compared to adjacent normal tissues (**Figure 11G**). Analysis of colon cancer cell lines (HCT116, SW480, LoVo) showed higher TIMP1 expression than the normal FHC cell line, with Western blot confirming strong TIMP1 immunoreactivity (**Figure 11H**).

TIMP1 overexpression significantly increased the migratory and invasive potential of HCT116 cells (141 vs. 94 cells in control), while TIMP1 knockdown reduced these behaviors (74 vs. 94 cells in control) (**Figures 11I–K**). These findings establish a clear correlation between TIMP1 expression and enhanced migratory/invasive characteristics in COAD.

Immunofluorescence (IF) analysis illustrated that TIMP1 exhibits a perinuclear localization associated with the Golgi apparatus and vesicles, showing a stronger immunohistochemical (IHC) signal in COAD when compared to normal tissues. While these observations are correlative in nature, elucidating the mechanistic roles of TIMP1 necessitates the implementation of functional perturbation studies and rescue experiments.

### 3.17 STAD validation: tissue RNA, WB, and Transwell migration

**Figure 12:**
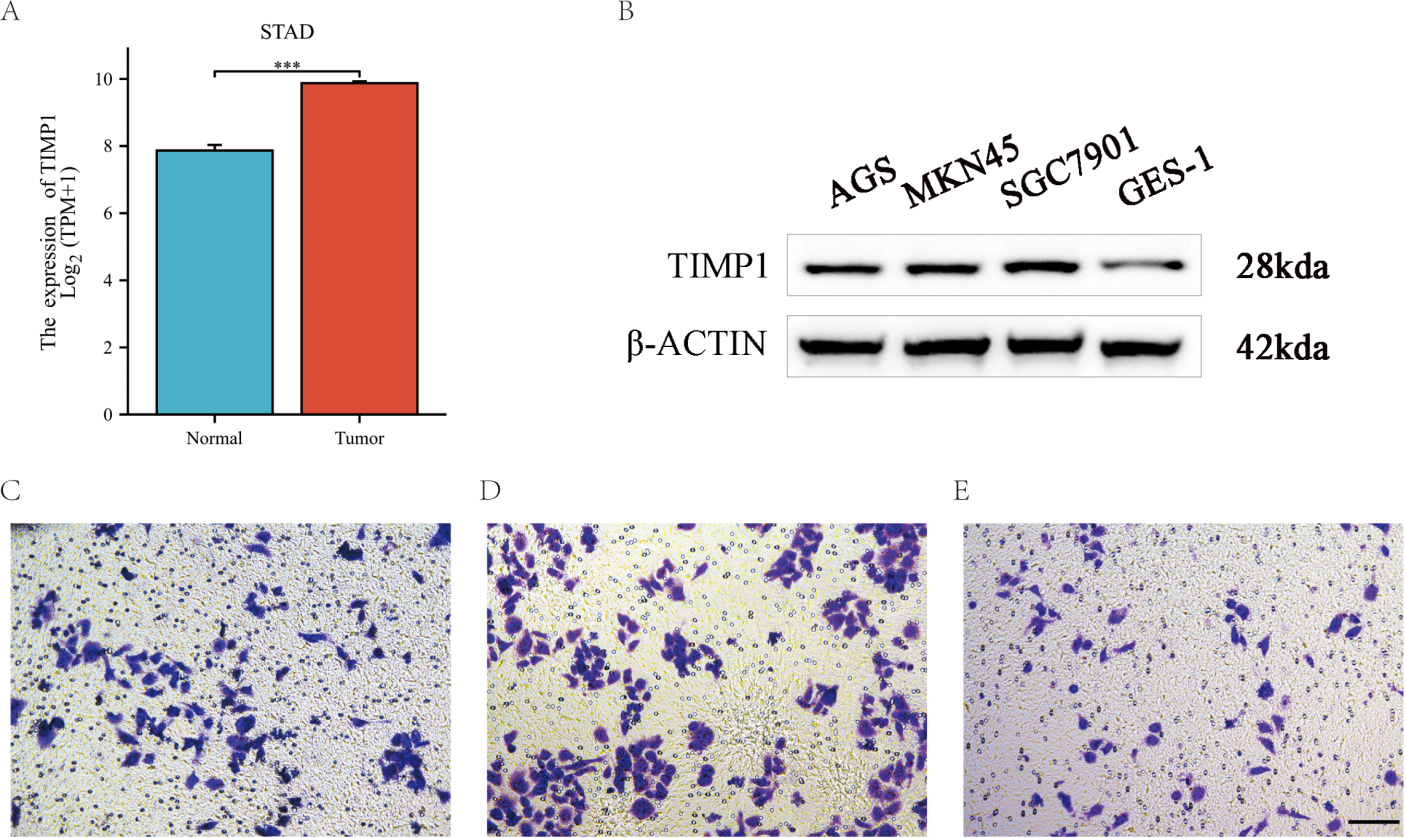
Independent gastric cancer validation (STAD): tissue RNA, Western blot, and Transwell assays. **(A)** Tissue RNA expression of TIMP1 in STAD vs adjacent normal stomach (TCGA). Effect size (Δ), sample sizes, and two-sided P/FDR are displayed in the panel. **(B)** Western blot of TIMP1 in GES-1 (normal) vs STAD cell lines (AGS, MKN45, SGC7901) with GAPDH/β-actin loading controls; densitometry normalized to GES-1 = 1.0 (n=3 biological replicates). **(C-E)** Transwell migration assays using the AGS gastric cancer (STAD) cell line under empty vector control **(C)**, TIMP1 overexpression (**D**, OE), and knockdown (**E**, KD). Representative micrographs and quantification (mean ± SD, triplicate inserts; ≥3 independent experiments). Group comparisons use one-way ANOVA with Tukey *post-hoc*; when multiple pairwise tests are conducted within a panel, BH–FDR correction is applied (q<0.05). “OE” refers to TIMP1 overexpression, and “KD” refers to TIMP1 knockdown.

Tissues from stomach adenocarcinoma (STAD) exhibited elevated levels of TIMP1 RNA when compared to adjacent normal tissues (**Figure 12A**). In cellular models, Western blot analysis demonstrated significantly higher TIMP1 protein expression in the AGS, MKN45, and SGC7901 cell lines in contrast to GES-1, with quantification achieved through densitometry and normalized against loading controls (n=3; **Figure 12B**).

**Table 1:** The 162 DEGs.

ID	logFC	adj.p	ID	logFC	adj.p	ID	logFC	adj.P
Lcn2	4.426833	1.87E-07	Igfbp4	1.386847	5.97E-05	Man2a1	1.068257	5.97E-05
Scgb3a1	3.573457	1.8E-06	Fgl2	1.379823	8.11E-05	Trf	1.067147	0.00015
Srpx	3.174053	8.22E-07	Ms4a6c	1.372203	2.27E-05	Entpd1	1.064897	6.77E-05
Apod	2.93936	1.87E-07	Cfh	1.37013	1.04E-05	Ang	1.061133	0.000248
P21	2.90413	7.52E-06	Cd163	1.367897	1.01E-05	Dpt	1.059867	0.000129
Prg4	2.841563	2.04E-07	C7	1.327863	0.000113	Npl	1.057273	9.94E-05
Vsig4	2.546533	2.69E-05	Mmp2	1.326757	2.51E-05	Tcf23	1.04312	0.000179
C4B	2.520847	5.47E-07	Il1r1	1.32198	4.93E-05	Ms4a6b	1.041977	0.000365
Ahsg	2.48108	5.47E-07	Ccdc80	1.310663	7.96E-05	Hal	1.039437	0.000136
Lrg1	2.329263	1.01E-05	Tmem176b	1.307993	2.69E-05	Irak3	1.03219	0.00027
Il33	2.22194	0.000102	Vcan	1.30393	0.000531	Comt	1.03206	0.000147
Mt2a	2.198567	5.15E-07	Mgst1	1.301093	0.000144	Htra3	1.02818	0.000172
Chil1	2.03028	5.13E-06	Pi16	1.29833	0.000255	Atg13	1.025433	0.00025
Gfpt2	2.025067	1.7E-06	Sdf2l1	1.298197	0.002044	Egfr	1.021097	0.00015
Ms4a6d	2.00103	1.8E-06	Myc	1.297217	0.000565	Cmklr1	1.02095	0.000116
Timp1	1.949763	0.001662	2210407C18Rik	1.283327	0.000292	Pgm3	1.020417	0.000359
Ms4a4a	1.903787	4.31E-05	Map3k6	1.274387	7.83E-05	Ctss	1.01468	0.000841
Mmp3	1.883503	2.27E-05	C1s1	1.27181	1.45E-05	Lect1	1.012673	0.000565
Mt1	1.8757	1.8E-06	Tnfaip6	1.260073	0.000831	Hpse	1.009887	0.000156
Cyp1b1	1.837917	5.82E-05	Fam107a	1.257447	0.001295	NESH	1.00501	0.00012
Ifi205	1.82827	4.76E-05	Ch25h	1.255007	0.00655	B4galt5	1.000683	0.00015
Syt12	1.825837	8.1E-06	Has2	1.231303	0.006966	3110040M04Rik	-1.00197	6.38E-05
Pi15	1.821953	5.75E-05	Fcgr2b	1.216343	2.79E-05	Lrtm1	-1.0062	0.00317
Mctp1	1.79773	2.63E-06	f-spondin	1.204743	0.000156	Cenpa	-1.03019	0.000331
Tmem176a	1.741583	1.35E-05	Slc3a2	1.1987	5.22E-05	Fam78a	-1.03716	0.000364
Gda	1.68032	7.52E-06	Tmem38b	1.186847	2.3E-05	Tgtp2	-1.03805	0.00383
Mmp19	1.678803	4.22E-06	St3gal1	1.17065	6.33E-05	Akr1c14	-1.04103	0.001838
Osmr	1.65507	7.35E-06	Acer2	1.167967	4.64E-05	Mest	-1.0414	0.000102
PAF-AH	1.642723	1.35E-05	Prkar2b	1.167207	0.001662	Ccnd1	-1.05053	0.00015
Hp	1.640473	2.51E-05	C1qa	1.16217	0.000151	Lsmem1	-1.05718	0.000107
Lbp	1.632953	1.69E-05	GEM	1.154227	5.22E-05	H2-Ea-ps	-1.0578	0.000292
C3	1.628533	1.93E-05	Emb	1.148613	0.000748	Phlda1	-1.07606	0.003007
Il4ra	1.614657	0.000359	Tgfbr2	1.147473	6.26E-05	AW112010	-1.08393	0.001049
Olfml2b	1.609247	2.51E-05	Gm15448	1.147127	9.94E-05	Fam81a	-1.08589	0.000135
Lrrc52	1.608347	0.000252	Nfil3	1.143647	0.037732	Bhlhe41	-1.09093	0.000718
Gpr133	1.589307	4.33E-05	Myot	1.142117	0.007599	Casq1	-1.1776	0.000129
Tmem100	1.582523	1.01E-05	Enc1	1.132167	0.000393	Ces1d	-1.17953	0.000717
Lyve1	1.581973	1.31E-05	Plek	1.119723	6.29E-05	Retnla	-1.18848	0.000385
Ppbp	1.56956	0.001662	Nxpe5	1.116757	0.002624	Kcnip2	-1.20106	0.000129
Fah	1.55508	0.000129	Ctla2a	1.115423	0.002283	Gm4841	-1.20838	0.000531
Mpzl2	1.549703	3.29E-05	Selp	1.112963	0.00042	Ciart	-1.21601	0.00015
Itih4	1.525557	0.000164	BGT1	1.11068	0.002012	Inmt	-1.25394	0.000863
Fetub	1.522037	0.000172	Cbr2	1.108867	0.000301	HLA-G	-1.25865	5.64E-05
H19	1.498037	5.92E-05	Htra4	1.10049	0.000613	Meig1	-1.29277	2.3E-05
AI607873	1.492573	4.76E-05	Tmtc1	1.09676	8.67E-05	Itgb6	-1.29855	2.49E-05
Clca1	1.47014	4.93E-05	Pgf	1.09401	6.77E-05	Scgb1c1	-1.40257	0.001497
Lox	1.465233	7.83E-05	Clec4d	1.092967	0.000678	Nrep	-1.44081	0.000915
Wfdc17	1.432497	1.35E-05	Mrap2	1.091883	0.000678	B3galt2	-1.53038	0.000342
Fkbp5	1.428513	4.49E-05	Ifi204	1.090073	0.000121	Aqp6	-1.57572	1.93E-05
F13a1	1.42101	2.3E-05	Mmp14	1.08916	0.000301	Aplnr	-1.59657	0.000129
Ifi202b	1.416833	4.76E-05	Igf1	1.083353	0.000359	BC023105	-1.61937	0.000152
Cyp2e1	1.411587	0.001263	SPRED2	1.080813	0.000292	Mylk4	-1.8746	1.8E-06
Tlr13	1.406413	6.3E-05	Errfi1	1.078263	0.002624	Lgi1	-1.93874	0.000297
Mrc1	1.39001	1.04E-05	Socs3	1.074793	0.000301	Fgf16	-2.05361	1.41E-05

Furthermore, Transwell assays revealed that overexpression of TIMP1 resulted in increased migration counts, whereas silencing TIMP1 expression led to a decrease in these counts when compared to vector controls (mean ± SD; analyzed using one-way ANOVA with Tukey’s *post-hoc* test; Benjamini-Hochberg false discovery rate correction applied for multiple comparisons; **Figures 12C–E**). It is important to note that these interpretations are correlational in nature; a deeper understanding of the mechanistic roles of TIMP1 necessitates functional perturbation studies and subsequent rescue experiments.

## 4 Discussion

In this study, we systematically characterized TIMP1 across cancers and cardiovascular disorders through an integrative multi-level and bioinformatics framework. Starting from tumor-bearing mouse heart transcriptomes, TIMP1 was identified as a hub gene correlatively associated with ECM remodeling, immune-response pathways and PI3K–Akt/MAPK signaling. Pan-cancer profiling demonstrated widespread TIMP1 upregulation, frequent genomic alterations, and associations with TMB, MSI, immune infiltration, and unfavorable prognosis, particularly in gastrointestinal cancers (2, 28).

Functional validation in colorectal and gastric models further confirmed that TIMP1 enhances migratory and invasive behaviors, supporting its role as a driver of malignant progression (29). These results are consistent with the established role of ECM regulators in shaping tumor aggressiveness and align with the broader concept that stromal–immune remodeling represents a key hallmark of cancer biology (30).

Our analysis highlights three dimensions of TIMP1 activity. At the tumor tissue level, TIMP1 correlated with CAFs, endothelial cells, and macrophages, and was enriched in the TGF-β–dominant immune subtype, reinforcing its involvement in stromal–immune crosstalk (31).

At the single-cell level, TIMP1 localized predominantly to myeloid subsets in colorectal cancer and fibroblasts in gastric cancer, with distinct pathway signatures (ROS/apoptosis versus EMT/angiogenesis), suggesting lineage-specific programs (32).

At the cardiovascular level, TIMP1 showed opposing regulation, being elevated in atherosclerosis with immune–lipid pathway enrichment but downregulated in heart failure with mitochondrial dysfunction. These findings not only reveal a context-dependent role for TIMP1 but also extend its implications beyond oncology into cardiovascular remodeling (3, 33). These cardiovascular signatures also resonate with mechanisms underlying cancer–heart disease comorbidities, where tumor-driven inflammation and metabolic stress accelerate vascular injury and cardiac dysfunction.

The context-dependent regulation of TIMP1 observed here may therefore represent a shared remodeling axis across malignancy and cardiovascular disorders. Compared with previous studies focusing on individual cancer types or isolated cardiovascular models, our work integrates pan-cancer, cross-disease, and functional perspectives, thereby providing a more comprehensive understanding of TIMP1 biology.

Several novel contributions emerge from this study. First, this is among the first attempts to place TIMP1 in a pan-cancer multi-level landscape, uncovering its consistent overexpression, prognostic relevance, and immune associations across diverse malignancies. Second, we extend the investigation to cardiovascular disorders, identifying a bidirectional regulatory pattern in atherosclerosis versus heart failure, which has not been systematically reported before. Third, our combined use of bulk, single-cell, and experimental validation provides convergent evidence that TIMP1 functions as both a biomarker and a potential mediator of tumor–immune–stroma interactions. These findings highlight TIMP1 as a candidate cross-disease biomarker at the interface of cancer biology and cardiovascular pathology.

Nonetheless, several limitations should be acknowledged. The majority of our findings are correlative, and TIMP1 should not be overinterpreted as a therapeutic target without causal or translational validation. Likewise, enrichment and GSEA results indicate pathway associations rather than mechanistic evidence, and signaling inferences should be interpreted cautiously to avoid attributing causality to correlation.

Although unified processing was applied, reliance on public datasets introduces batch effects, heterogeneous clinical annotations, and potential residual confounding in multi-cancer survival analyses. In particular, TCGA-based associations may be influenced by unadjusted clinical covariates such as age, sex, and tumor stage, and we acknowledge this as an additional limitation.

In our experimental validation, some *in-vitro* assays were based on limited biological replicates (typically n = 3), and independent external datasets were not available for additional validation, which may affect the robustness and generalizability of the findings. Finally, the cardiovascular analyses were restricted to existing atherosclerosis and heart-failure cohorts, which may not capture the broader spectrum of cancer-related cardiac remodeling. These limitations underscore the need for expanded biological replication, *in vivo* perturbation studies, independent validation cohorts, and longitudinal clinical datasets.

Future work should focus on several directions. Mechanistic dissection of TIMP1 receptor interactions in fibroblasts and myeloid cells, combined with spatial and temporal transcriptomic profiling, will help delineate downstream pathways.

Functional models using *CRISPR*-based perturbation and targeted rescue strategies could test whether TIMP1 directly drives immune evasion or stromal activation. Clinically, incorporating TIMP1 into immune-subtype and drug-response frameworks may guide patient stratification for epigenetic modulators or stroma-targeted therapies. Given its divergent roles in atherosclerosis and heart failure, TIMP1 also merits investigation as a biomarker for cancer–cardiovascular comorbidities, where it may serve both as a prognostic marker and a therapeutic target. Together, these directions will establish TIMP1 as a bridge linking tumor progression, immune regulation, and cardiovascular remodeling.

## 5 Conclusion

In summary, our multi-layered analyses establish TIMP1 as a context-dependent regulator bridging cancer biology and cardiovascular pathology. Its consistent overexpression in digestive cancers, association with stromal–immune programs, and functional role in promoting invasion highlight its potential as a prognostic, diagnostic, and therapeutic biomarker. At the same time, its inverse patterns in atherosclerosis and heart failure emphasize the need for disease- and tissue-specific interpretation. Together, these findings position TIMP1 as a promising target at the interface of oncology and cardio-metabolic research.

## Data Availability

The original contributions presented in the study are included in the article/supplementary material. Further inquiries can be directed to the corresponding authors.
